# Minutes of the Fourth Annual Meeting of the Ohio Dental College Association

**Published:** 1856-04

**Authors:** 


### MINUTES OF THE FOURTH ANNUAL MEETING OF THE OHIO DENTAL COLLEGE ASSOCIATION.

The Association met according to adjournment, in the College Building, at 2 P. M., of Monday, February 18th, 1856.

Present, Drs. JAMES TAYLOR, TAFT, ULREY, WATT, H. R. SMITH, J. B. SMITH, KEELY and BONSALL.

The minutes of last year were first read, after which, Dr. Bonsall was appointed to fill the vacancy in the examining committee, occasioned by the absence of Dr. T. R. Willard, when so as to give time to examine the candidates for graduation, on motion, the Association adjourned till 3 P. M. to-morrow.

SECOND DAY.

Met at 3 P. M., according to adjournment. Present, Drs. Goddard, Griffith, James Taylor, H. R. Smith, Taft, Watt, Keeley, Ulrey, Bray and Bonsall.

The following report was received from the building and finance committee:

*To the Ohio Dental College Association:—*

GENTLEMEN: The committee appointed in 1854, to superintend the erection of the College building, and to attend to its financial affairs, beg leave to offer the following in addition to their last year's report.

They have not been able as yet, to secure the Four Thousand dollars on a mortgage at 10 per cent. as expected, and which was then promised. This disappointment has very much embarassed the committee, and has forced them to pay a heavier rate of interest on the three thousand dollars due to Messrs. Taylor and Talbott, and a still larger rate on the seventeen hundred and twenty-three dollars and eighty-three cents then borrowed.

A considerable portion of the amount which was then, and which has since became due, has not been paid in as was expected. These circumstances render the financial condition of the College not as good as we should be glad to report.

The committee would however, present the following account current for the year, and up to the 1st of April proximo—showing the condition of the Institution, hoping the Association may devise some means by which it may be relieved from future embarrassment.

The indebtedness is as follows, viz:

| | |
|---|---|
| Due to Taylor and Talbott, - - - | $3,000,00 |
| " for money borrowed, - - - - | 1,723,83 |
| Interest paid on the $3000, - - - | 334,94 |
| " " " " 1,723,83, - - - | 310,27 |
| | $5,369,04 |

The receipts are as follows:

| | |
|---|---|
| On account of Stock, - - - | $377,40 |
| " " back Rent, - - - | 84,10 |
| " " Int. paid by Dr. Taylor on these sums in his hands, | 26,96 |
| Rent paid by the Faculty, - - | 600,00 |
| | $1,088,46 |

Leaving a balance still due on the 1st April next, of $4,280,58

Respectfully,

JAMES TAYLOR,

CHAS. BONSALL, } *Committee.*

After the reading of the report, Drs. Ulrey, Taft and H. R. Smith were appointed to audit the accounts and report at next meeting.

On motion, Drs. C. B. Chapman of Cincinnati, J. B. Miner of Milwaukee, Wis., Saml. Griffith of Louisville, Ky., and James O. Martin of Franklin, Ind., having each subscribed for a share of Stock, were balloted for and unanimously elected members of the Association and signed the Constitution.

On motion of Dr. Taft, it was

*Resolved*, That the Trustees of the College property be requested to have prepared by the next annual meeting, proper Certificates of Stock, to be duly registered, and that the Stockholders be requested to bring or send in their present certificates at that time, to be exchanged for new ones, and on motion the Trustees were authorized to adopt a style of certificate such as they may think suitable.

The death of two members of our Association, since our last meeting, being announced, it was, on motion of Dr. Watt,

*Resolved*, That in the death of Drs. S. Fithian, of St. Louis, Missouri, and T. L. Waring, of Zanesville, Ohio, our Association has lost two of its most worthy members and the Dental Profession two of its bright ornaments.

*Resolved*, That we tender our most sincere sympathies to the bereaved and afflicted families and friends of the deceased.

The following report was received from the Faculty, and accepted, viz:

The Faculty of the Ohio College of Dental Surgery would present the following report:

The changes recommended by the Association at our last yearly meeting have all been consummated. At as early a day as practicable, the Trustees were called together, when they created a chair of Dental Chemistry and Metallurgy, and confirmed the nomination of Dr. George Watt to the same.

Professor T. Wood M. D., (as expected,) handed in his resignation, and the Faculty unanimously recommended to the Trustees C. B. Chapman, M. D., who was duly elected Professor of Anatomy and Physiology.

The Trustees also changed the title of the Chair of Principles and Practice of Dental Surgery to that of Institutes of Dental Science, and elected James Taylor, M. D. to the same.

The Faculty regard these changes in the chairs of the Institution such as fully to meet the wants of Dental education, and think that the present arrangement affords as full scope for instruction in each department as could be desired.

They have aimed to make their instruction as full, comprehensive and practical as possible.

They are happy to report a gradual increase in the size of the class, and that they have cause for encouragement in the assiduous manner in which they have devoted themselves to their studies.

We would also gratefully refer to the courteous treatment we have received personally from the class, and the general harmony which prevailed among them.

In presenting a larger number of candidates for graduation than heretofore, the Faculty feel that it is, in part, at least, the effect of the strict requisitions required. The seal of an Institution to be desired must be made of intrinsic value.

Three of our candidates for graduation are already regular graduates of Medicine. Four have been in the practice of Dentistry for from four to ten years, and three have attended two full courses in this Institution.

They would also report the following as the amount of operations performed, during the present session, in the Infirmary of the Institution:

| | |
|---|---|
| No. of teeth filled with gold, | 180 |
| " " with other materials, | 50 |
| " " extracted, | 262 |
| " of patients having Salivary Calculus removed, | 22 |
| " Pivot teeth inserted, | 5 |
| " Diseased antrums treated, | 1 |
| " Full sets inserted, | 9 |
| " Upper sets, | 2 |
| " Partial sets | 7 |
| " Operations repaired, | 6 |

The following rules for the government of the Infirmary, during the summer, were adopted by the Faculty:

*Rules and Regulations of the Infirmary of the Ohio College of Dental Surgery.*

1. The Infirmary shall be under the general control of the Faculty.

2. Its special management, except during the College sessions, shall be delegated to a member of the graduating class, each year, who shall be chosen by the faculty, on examination, from members of the class, who may desire the situation,

3. Said incumbent shall keep the Infirmary open during the usual office hours—shall keep a record of all operations performed, diseased conditions treated, &c., according to the instructions of the Faculty, and, in general, shall endeavor to promote the cause of Dental Science, and advance the interests of the Institution.

The prices for Infirmary operations shall be as follows:

| | |
|---|---|
| For filling, net profit, | 25 to $ 50 |
| " Extracting, | 00 |
| " Pivot tooth, | 1 00 |

| | | |
|---|---|---|
| For | Plate teeth, partial sets, on gold, per tooth, | 2 00 |
| " | Full sets on gold, plain, - - - | 50 00 |
| " | " " " socket rim, - - - | 60 00 |
| " | Partial sets, on silver, per tooth, - - | 1 50 |
| " | Full sets, " " - - | 25 00 |
| " | Removing tartar, &c., - - - | ,50 to 1 00 |

5. The incumbent may occupy rooms, Nos. 1, 2, and 3, for office, sleeping room, and laboratory.

6. A supervisory committee of three shall be appointed each year by the Faculty, which shall have all the power of the Faculty, in controlling the Infirmary, except during the College sessions.

7. The incumbent shall be required to report to the supervisory committee at the close of every fourth week, and to pay, to the same, one-half the net profits of the Infirmary practice, to be appropriated toward furnishing the College with furniture, apparatus, &c.

8. Students of Dentistry who have attended one course in the College, those who have taken the tickets for a future course, and matriculants, whose preceptors are members of the College association, shall be entitled to witness the operations in the Infirmary, and to operate, when needed, by arrangement with the incumbent and committee.

9. Said imcumbent may, for violation of these rules, malpractice, or immorality, be dismissed at any time by the Faculty.

| | | |
|---|---|---|
| *Supervisory Committee for* 1656. | James Taylor,<br>J. B. Smith,<br>Geo. Watt, | *Infirmary Committee.* |

The auditing committee handed in the following report, which was accepted and the committee discharged.

"Your Committee to audit the Treasurer's Report would respectfully submit that they have examined the accounts and find the same to be correct..

J. Taft,<br>
J. P. Ulrey,<br>
H. R. Smith.

The Association now went into an election of officers for the ensuing year, which resulted as follows:

*President*—Dr. James Taylor; 1*st Vice President*—Dr. J. Taft; 2*d Vice Pressdent*—Dr. J. P. Ulrey; *Secretary*—Dr. Charles Bonsall; *Treasurer*—Dr. J. B. Smith.

Dental Examiners, { " E. Collins, Connersville, Ia.

" B. Strickland, Cleveland, O.

" J. B. Miner, Milwaukee, Wis. }

Medical Examiners, { " James Fishback, Shelbyville, Ia.

" C. G. Goodrich, Oxford, O. }

Adjourned to meet at 8 o'clock, A. M. on Thursday.

THIRD DAY.

Met according to adjournment, with a full attendance of members.

The following resolution, moved by Dr. Bonsall, and seconded by Dr. Ulrey, was unanimously carried.

*Resolved*, That the amount of rent to be paid by the Faculty be strictly the 6 per cent. on the amount of stock taken and authorized to be sold in addition to the taxes and insurance, as now paid.

The business of the Association now being through with, they adjourned to meet at the same place, at 2 o'clock of Monday, Feb. 23, 1857.

CHARLES BONSALL, *Secretary.*

The Secretary would also report that the commencement exercises took place on the evening of Wednesday the 20th, and were well attended, there being many members of the profession from a distance, as well as our own citizens, in attendance, and an evident increased interest in the success of the Institution being shown.

The valedictory address was delivered by Prof. Taft, whose subject was "The Progress of the Age."

The conferring the Degrees and advice to the graduating class was by the Rev. Samuel W. Fisher, D. D., and a reply by C. H. James, one of the graduates.

The following gentlemen, all of whom passed a satisfactory examination, received the Diplomas:

Edmund Osmond, Port Jefferson, Ohio.
A. G. Stipher, Canton Illinois.
John F. Mercer, Canada West.
A. D. Nevius, Lexington, Mississippi.
William G. Drake, Aberdeen, do.
Franklin Grimes, Fayette, Mo.
John R. James, Ky.
Charles H. James, Cincinnati, O.
Franklin Bell, do.
Thomas F. Davenport, do.

